# Polysulfone Membranes Embedded with Halloysites Nanotubes: Preparation and Properties

**DOI:** 10.3390/membranes10010002

**Published:** 2019-12-25

**Authors:** Nagla Kamal, Viktor Kochkodan, Atef Zekri, Said Ahzi

**Affiliations:** 1 College of Science and Engineering, Hamad Bin Khalifa University (HBKU), P.O. Box 34110 Doha, Qatar; sahzi@hbku.edu.qa; 2 Qatar Environment and Energy Research Institute (QEERI), Hamad Bin Khalifa University (HBKU), P.O. Box 34110 Doha, Qatar; azekri@hbku.edu.qa

**Keywords:** nanocomposite membranes, polysulfone, halloysite, porous structure, mechanical properties

## Abstract

In the present study, nanocomposite ultrafiltration membranes were prepared by incorporating nanotubes clay halloysite (HNTs) into polysulfone (PSF) and PSF/polyvinylpyrrolidone (PVP) dope solutions followed by membrane casting using phase inversion method. Characterization of HNTs were conducted using scanning electron microscopy (SEM), transmission electron microscopy (TEM), energy-dispersive X-ray spectroscopy (EDS), X-ray diffraction (XRD), and thermogravimetric (TGA) analysis. The pore structure, morphology, hydrophilicity and mechanical properties of the composite membranes were characterized by using SEM, water contact angle (WCA) measurements, and dynamic mechanical analysis. It was shown that the incorporation of HNTs enhanced hydrophilicity and mechanical properties of the prepared PSF membranes. Compared to the pristine PSF membrane, results show that the total porosity and pore size of PSF/HNTs composite membranes increased when HNTs loadings were more than 0.5 wt % and 1.0 wt %, respectively. These findings correlate well with changes in water flux of the prepared membranes. It was observed that HNTs were homogenously dispersed within the PSF membrane matrix at HNTs content of 0.1 to 0.5 wt % and the PSF/HNTs membranes prepared by incorporating 0.2 wt % HNTs loading possess the optimal mechanical properties in terms of elastic modulus and yield stress. In the case of the PSF/PVP matrix, the optimal mechanical properties were obtained with 0.3 wt % of HNTs because PVP enhances the HNTs distribution. Results of bovine serum albumin (BSA) filtration tests indicated that PSF/0.2 wt % HNTs membrane exhibited high BSA rejection and notable anti-fouling properties.

## 1. Introduction

Currently, polymeric membranes, due to their low cost, ease of processing, and chemical stability [1,2,3], are widely utilized in numerous membrane based processes, such as gas separation [4,5], pervaporation [6,7], desalination, and water treatment [8,9,10]. Polysulfone (PSF) is one of the most popular and broadly used polymers for the fabrication of microfiltration (MF) and ultrafiltration (UF) membranes [11,12]. In addition, PSF is used for the preparation of porous supports for nanofiltration (NF), reverse osmosis (RO), and forward osmosis (FO) membranes [11,13]. PSF has good chemical, thermal, and mechanical properties as well as an excellent film forming potential and tolerance to a wide range of pH [12,14]. However, the main shortcoming of PSF as a membrane material is the hydrophobic nature of this polymer, which causes PSF membrane fouling and relatively low tensile strength [12,15]. Mechanical properties, such as elastic modulus and yield stress, are important characteristics for polymeric membranes used for pressure-driven membrane processes [16]. Under operating pressure, the membranes undergo physical compaction that causes an irreversible loss of water flux [17]. Also, polymers with low tensile strength are more sensitive to failure under operating pressure or hydraulic stresses imposed by membrane backwashing procedure [18]. To improve the membranes’ elastic modulus and yield stress, the incorporation of inorganic materials that have excellent mechanical and functional properties to membrane polymeric matrices can be used [19].

Inorganic nanomaterials, such as zeolite, graphite, graphene oxide (GO), and silica, have been incorporated into the membrane polymer matrix to enhance its performance and properties [20,21,22,23]. One of the challenges associated with the use of inorganic nanomaterials is their tendency for agglomeration in the polymer matrix [24]. The amount of nanomaterial incorporated into polymer should be optimized to enhance interactions at a polymer-filler interface and minimize agglomeration of the nanomaterial [25]. Studies suggested that using one-dimensional (1D) and two-dimensional (2D) inorganic nanomaterials results in a more even distribution within the membrane matrix and decreases leakage of the nanomaterials from the membrane [24,26].

Among the 1D nanomaterials that have been used in mixed-matrix membranes are carbon nanotubes (CNTs), which gained interest in the synthesis of novel nanocomposite membranes for water treatment [27]. Incorporation of CNTs into the polymer matrix has shown promising results in improving membrane anti-fouling behavior, mechanical strength, membrane rejection, and water flux [28,29,30]. However, the high cost of CNTs is one of the main factors that limits CNTs application in membrane preparation [31]. Additionally, some studies show the toxic effect of CNTs on human health [32] if they leaked out from the membrane to the treated water. 

**Figure 1:**
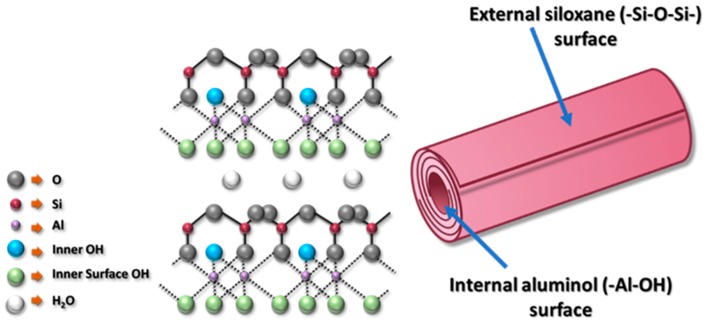
Schematic representation of halloysites (HNTs) and its chemical structure consisting of rolled sheets of external siloxane and internal aluminol.

Halloysite (HNTs) is a type of 1D nano-sized clay minerals composed of aluminosilicate [33]. HNTs are naturally existing 1:1 multi-walled inorganic nanotubes built of tetrahedral (SiO) and octahedral (Al–OH) sheets, which has the chemical formula of Al_2_(OH)_4_Si_2_O_5_·nH_2_O [34]. It was shown that HNTs possess a combination of unique characteristics, such as tubular nanostructure, high aspect ratio, natural abundance, availability of functional groups, excellent biocompatibility, and strong mechanical strength [35,36,37]. The length of HNTs varies from 200 to 2000 nm with its internal and external diameter ranging from 10 to 40 nm and 40 to 70 nm, respectively [35]. The difference between most clays and HNTs is that the aluminol sheets are found inside the tubular structure of HNTs. Figure 1 represents a schematic diagram of HNTs nanotube and its chemical structure. 

In recent decades, HNTs have attracted remarkable scientific attention due to their exceptional physicochemical properties and have been employed as a filler in polymeric materials, a guest loading carrier for controlled release of the guest molecules, and as an absorbent for pollutant removal from water [38,39].

**Figure 2:**
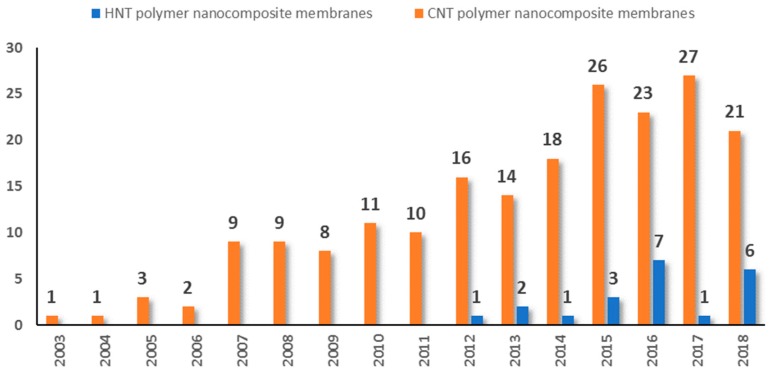
Comparison of the annual number of scientific publications on the carbon nanotubes (CNTs) and HNTs nanocomposite membranes since 2003 (information obtained by the author from Scopus document search website by using keywords: CNTs, HNTs, polymer, membrane).

Even though research on HNTs-polymer based membranes in water treatment applications are starting to grow since the last few years, there is still a paucity of studies on HNTs/polymer-based membranes compared to research conducted on CNTs/polymer-based membranes. Figure 2 shows a comparison of the annual number of scientific publications on the CNTs and HNTs composite membranes.

Mu et al. [40] prepared L-DOPA (L-DOPA is a mixture of hormone and amino acid produced naturally by many plants and animals [41]) functionalized HNTs (LDPHNT) and blended them in different ratios with cellulose acetate (CA) polymeric membrane. It was observed that the introduction of LDPHNT to CA membrane resulted in an increase in the number and size of the membrane’s surface pores, higher hydrophilicity, notable enhancement in mechanical properties, and a better anti-fouling performance for CA/LDPHNT hybrid membranes [40]. Zhu et al. [42] modified HNTs with poly (sodium-p-styrenesulfonate) (PSS) to be sandwiched between two layers of porous reduced graphene oxide (PRGO) and used it as an upholder to expand the interlayer space between adjacent PRGO sheets. PRGO/HNTs-PSS mixtures of different concentrations were prepared and immobilized onto a PAN-based membrane surface using an in-situ evaporation technique. It was shown that the pure water flux of the prepared nanocomposite membranes was improved due to the sandwiched structure of the additive, which provides more pathways and facilitates the passage of water [42]. Hebbar et al. [43] fabricated nanocomposite hollow fiber membranes using the dry–wet spin method. Their membranes were composed of different loading of HNTs that were chemically modified with poly (m-aminophenol) (PHNTs) and added to the polyetherimide membrane matrix. The study showed that the addition of 2 wt % of PHNTs increases both membrane porosity to 65.8%, reduce contact angle to 64.8°, and increase surface energy to 102.6 mN/m. As a result, the pure water flux increased to 104.9 L/m^2^h, and flux recovery after filtration of dyes solutions reached up to 90.3%. In addition, the rejection of both Reactive Red-120 and Reactive Black-5 dyes were found to reach 97% and 94%, respectively [43]. Ibrahim et al. [44] described the use of tannic acid (TA) to modify HNTs through one step self-polymerization. The modified HNTs (THNTs) were then incorporated into PSF with a PVP dope solution to fabricate loose hollow fiber NF membranes. In the study, it was observed that the number of macrovoids elevated in both sides of the membranes as the concentration of THNTs increased. Their membranes exhibited a reduction in water contact angle measurements as THNTs loading increased. It was also shown that the membrane with 0.36 g of THNTs exhibited increased porosity, surface charge, highest pure water flux of 92 L/m^2^h, and a rejection of >99% and >97.5% for reactive black 5 and reactive orange 16 dyes, respectively. Ghanbari et al. [45] prepared FO substrates composed of PSF with varying concentrations of HNTs (0.25, 0.5, 1.0 wt %) and examined the influence of HNTs on morphology and performance of thin film nanocomposite (TFN) FO membranes. It was observed that hydrophilicity, mean pore radius, total porosity, and pure water flux increased with increasing HNTs contents. It was stated that the thickness of the FO substrate was reduced when elevating HNTs contents. The study also showed that the defects of enlarged voids and presence of HNTs observed on the surface of the substrate could affect the integrity of the polyamide layer that will be created on top of the substrate [45]. Swapna et al. [46] incorporated HNTs modified with telechelic polyetheramines into PSF membranes. It was found that at low HNTs loading, the porosity of the neat PSF membrane was higher than PSF/HNTs composite membranes. The thermal stability of composite membranes was found to be decreased due to the existence of aliphatic chains found in polyetheramines HNTs modifier, while the hydrophilicity was enhanced with increasing HNTs loading. Water uptake of composite membranes decreased when adding up to 5 wt % of modified HNTs, which was ascribed to a decrease in the free volume and to increase in tortuosity in the membrane matrix [46]. 

Despite the great contribution of the above-mentioned studies on inclusion of HNTs into polymeric membrane matrixes, previous studies used raw or modified HNTs embedding with other membrane polymers [40,42,43,47,48,49,50,51,52,53,54,55,56,57,58,59,60,61,62,63,64,65] modified HNTs with PSF [46,66], raw HNT with PSF/PVP matrix for the preparation of porous substrate for thin film nanocomposite membranes [45,67] or used other membrane fabrication methods, such as the electrospinning technique [68,69]. 

The purpose of this study is to provide better insight into the effect of incorporation of raw HNTs into PSF membranes, particularly to gain a better understanding of how HNTs interact with a PSF polymer matrix. In addition, the study intended to investigate how different HNTs loadings affect morphology, porous structure, and performance with special focus on mechanical properties of PSF/HNTs nanocomposite membranes casted both with and without PVP additive. 

## 2. Materials and Methods 

### 2.1. Materials

PSF pellets (average molecular weight (Mw) ~35,000), *N*,*N*-dimethylacetamide (DMA, purity ≥99%), polyvinylpyrrolidone (PVP, average Mw 10,000), HNT (Mw 294.19), hydrochloric acid (37 wt %), Bovine Serum Albumin (BSA, 69 KDa) were obtained from Sigma-Aldrich^®^ (St. Louis, MO, USA). Sodium hydroxide was purchased from Fisher Scientific^®^ (Bishop Meadow Road, Loughborough, UK). Millipore deionized water (DI) was used to prepare all aqueous solutions. 

### 2.2. Methodology

Preparation of PSF and PSF/HNT nanocomposite membranes were done using the following steps. A dope solution containing 16 wt % PSF and 84 wt % DMA was mixed at 25 °C for 24 h using a magnetic stirrer, allowing PSF to be completely dissolved in DMA and form a homogenous dope solution. Another dope solution was prepared the same way but with the addition of 1.0 wt % PVP to the PSF/DMA solution. Membrane casting solutions have been prepared by adding different HNT loadings of 0.0, 0.1, 0.2, 0.3, 0.5, 1.0, 2.0, 5.0 wt % to PSF/DMA or PSF/DMA/PVP solutions. Each mixture was sonicated at 25 °C for 30 min using a Q500 sonicator probe (Thomas Scientific, Swedesboro, NJ, USA) with maintaining continuous stirring using labForce digital hotplate stirrer (Thomas Scientific, Swedesboro, NJ, USA) to further improve dispersion of HNTs in the dope solution. The casting solution was then degassed in a water bath at 25 °C for 30 min to remove air bubbles before casting. The membrane samples were cast using the phase inversion method by employing a flat sheet Labcoat Master membrane casting system (PHILOS, Gyeonggi-do, Korea). The prepared casting solutions were poured on a clean, dry glass plate, and cast at room temperature using a casting knife of a 200 μm gap height with a casting speed of 2.5 m/min. Then, the glass plate with cast membrane film was immersed into a coagulation bath filled with deionized (DI) water at room temperature and was left until the membrane was detached from the glass plate. The membrane was then washed and kept for 24 h into a plastic container that contained DI water to allow traces of the solvent to be removed. Membranes samples were then labeled according to the type of membrane matrix and HNTs incorporated concentration. Table 1 shows the composition of the prepared membranes.

### 2.3. Characterization of HNTs and PSF/HNTs Membranes

#### 2.3.1. Characterization of HNTs

HNTs structure and morphology was investigated by scanning electron microscopy (SEM) imaging using FEI Quanta FEG 650 FE-SEM (FEI, Hillsboro, OR, USA) at 5 kV. Elemental composition of HNTs was analyzed using elemental analyzer Bruker Quantax EDS (Billerica, MA, USA) at 15 kV. The internal tubular structure, internal and external dimensions of HNTs were evaluated by using transmission electron microscopy (TEM) with FEI TalosF200X TEM (FEI, Hillsboro, OR, USA) at 200 kV. The crystalline phase of NHTs was analyzed using Bruker D8 Advance X-ray diffractometer (Billerica, MA, USA) with Cu Ka radiation (λ = 1.5418 Å) and instrument settings of 40 kV and 40 mA. The scan range was set from 3° to 100° with step size at 0.010°. Thermal stability and degradation of HNTs were measured under a nitrogen atmosphere using Discovery TGA Operator supplied by TA Instruments (TA Instruments, New Castle, UK). The operating temperature was in the range of 0–800 °C at 10 °C/min step. 

#### 2.3.2. Characterization of PSF/HNTs Membranes 

##### Morphology, Pore Size, and Porosity 

Membranes’ surface and cross-section morphologies were studded by a field emission scanning electron microscopy (FE-SEM) technique conducted using FEI Quanta FEG 650 (FEI, Hillsboro, OR, USA) with a set vacuum condition at 3 kV. Before imaging, liquid nitrogen was utilized to prepare cross-section of the membrane samples before their coating with 3 nm gold layer.

Total porosities of the prepared membranes were determined by using the gravimetric method [70]. Three samples were taken from each membrane, and the average porosity was determined as follows: (a) membrane samples were cut into circle shapes having a diameter of 46 mm and then dried in an oven for 24 h at 50 °C before weighing their mass ($w_{d}$); (b) the dried membranes samples were immersed in DI water at 25 °C for another 24 h; (c) the membrane samples were taken from water and wiped gently using filter paper before weighing their wet mass ($w_{w}$). 

Calculation of total membrane porosity (ε) was conducted using Equation (1) [11].

(1) $$\varepsilon =\;\frac{w_{w}-w_{d}}{A\times l\times \;\rho }\times 100$$

where $w_{w}$, $w_{d}$ represent the wet and dry masses of the membrane sample, respectively, *ρ* is the density of DI water at 25 °C, *A* is the membrane surface area (m^2^), and *l* is the membrane thickness (m).

The Guerout–Elford–Ferry equation [52] below (Equation (2)) was used to calculate the average membrane pore size:

(2) $$r_{m}=\sqrt{\frac{\left(2.9-1.75\varepsilon \right)8\eta lQ}{\varepsilon A\Delta P}}$$

where ε is the total porosity, η is the viscosity of DI at 25 °C, *Q* is the permeate volume (m^3^), *A* is the membrane sample surface area (m^2^), *l* is the membrane thickness (m), and ΔP is the operating pressure (1.0 bar).

##### Hydrophilicity 

Measurements of the water contact angle at the membrane surface were conducted using a KRUSS DCA-25 contact angle goniometer (KRÜSS GmbH, Hamburg, Germany) with a 1.5 µL DI water droplet size. The contact angle of every membrane sample was measured at room temperature (25 °C) in 3 different locations on the membrane surface.

##### Mechanical Analysis

**Figure 3:**
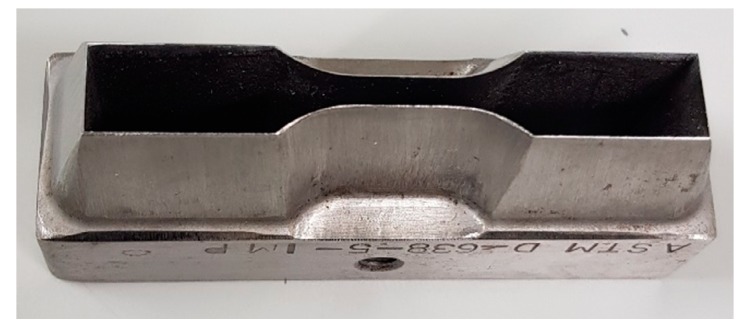
Dog-bone mold used to cut membrane samples used for dynamic mechanical analysis test.

Characterization of the mechanical properties (stress-strain relationship and elastic modulus) of membrane samples were investigated at room temperature using dynamic mechanical analysis Q-800 supplied by a TA Instrument (TA Instruments, New Castle, UK). The samples were cut by a dog-bone-like mold (Figure 3) to ensure the stress was focused on the middle of the sample’s length. The membrane samples were gripped on the testing fixture and stretched with a controlled constant displacement rate of 100 µm/min under tensile load until the samples breakage. The obtained stress–strain curves were used to determine the elastic modulus and yield stress values using TA universal analysis software (TA Instruments, New Castle, UK). The elastic modulus was determined from the slope of the linear part of the stress–strain curve, and the yield stress was determined from the onset point of plastic deformation (onset of yielding) and considered to be the stress at the stress–strain curve that corresponds to 3% strain.

### 2.4. Membranes Performance

**Figure 4:**
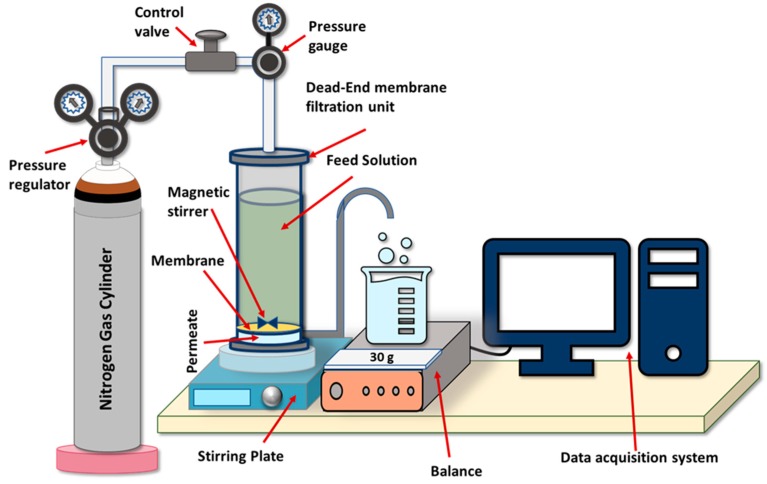
Schematic representation of a dead-end filtration system.

Membrane samples stored in DI water were thoroughly washed with DI water before using in a filtration test to remove any impurities. Pure water flux of the fabricated membranes was measured using a dead-end HP4750X stirred cell (Sterlitech, Kent, WA, USA). Nitrogen gas was used to pressurize feed solution inside the cell. Figure 4 shows a schematic representation of the bench scale filtration system used in the study.

The permeate flux (*J*) L/m^2^h (LMH) through the membrane was calculated as [71]:

(3) J=Q/(A×T)

where *A* is the effective membrane area (m^2^), *T* is the filtration time (h), and *Q* is the permeate volume (L).

The same dead-end filtration set-up was used to filter a 100 mg/L BSA solution prepared in DI water. BSA was used as a protein fouling model to investigate the anti-fouling and rejection capacity of the prepared membranes. BSA rejection (*R*) of membranes was calculated using Equation (4) [44].

(4) $$\%\;R=\left(1-\frac{C_{p}}{C_{f}}\right)\times 100$$

where $C_{p}$ and $C_{f}$ are corresponding BSA concentrations of permeate and feed, respectively. BSA solutions’ concentrations were evaluated by measuring the optical densities of BSA solutions at 220 nm by using a Shimadzu spectrophotometer (Shimadzu, Kioto, Japan).

Anti-fouling properties of cast membranes were determined by flux recovery ratio (FRR):

(5) $$\%\;FRR=\left(\frac{J_{2}}{J_{1}}\right)\times 100$$

where *J*_1_ and *J*_2_ are initial DI water flux and final DI water flux before and after 30 min of BSA filtration, respectively. A 100 mg/L BSA solution was used in this study, and all filtration experiments were conducted at room temperature (25 °C). 

## 3. Results and Discussions

### 3.1. HNT Characterization

#### 3.1.1. Microstructure of HNT

**Figure 5:**
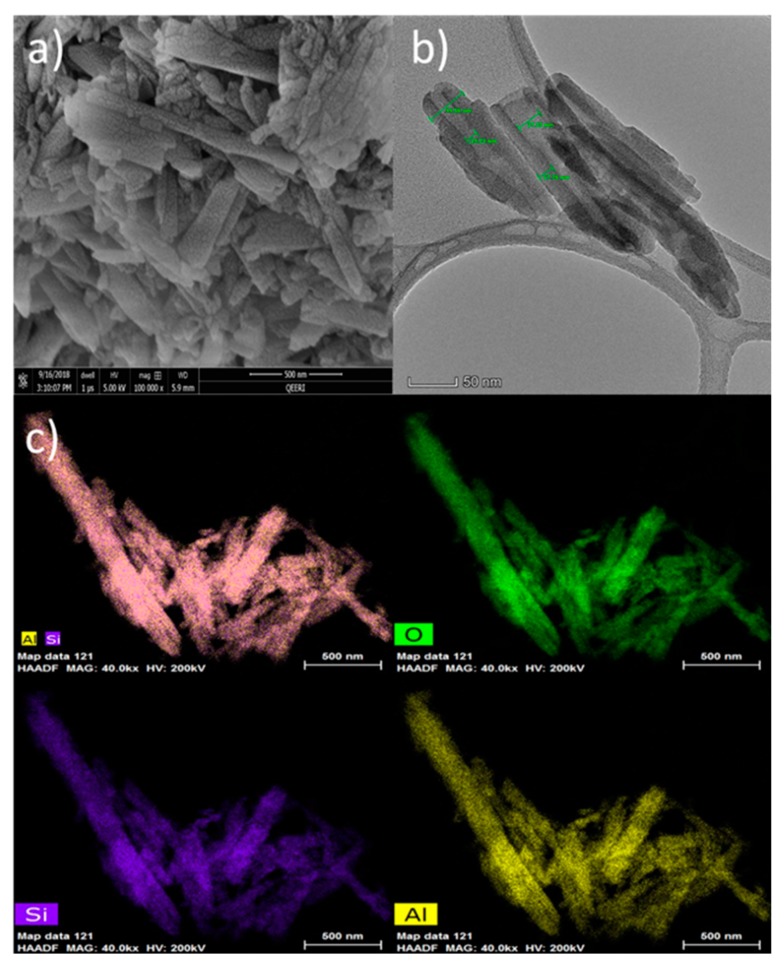
Raw HNTs characterization using (**a**) SEM, (**b**) TEM, and (**c**) EDS elemental mapping.

SEM and TEM images in Figure 5a,b show the tubular structure of HNTs samples with a well-defined empty and open-end lumen. The outer diameter for HNTs ranges from 44.6 to 45.8 nm and the inner diameter from 10.6 to 27.2 nm with a thickness of about 17.8 nm. Scanning transmission electron microscope (STEM) in combination with a high-angle annular dark-field imaging (HAADF) technique was used for elemental mapping of HNTs samples. As seen in Figure 5c, Si, Al, and O elements are uniformly distributed through the HNT nanoparticles.

#### 3.1.2. HNTs Chemical Composition and Crystallinity

**Figure 6:**
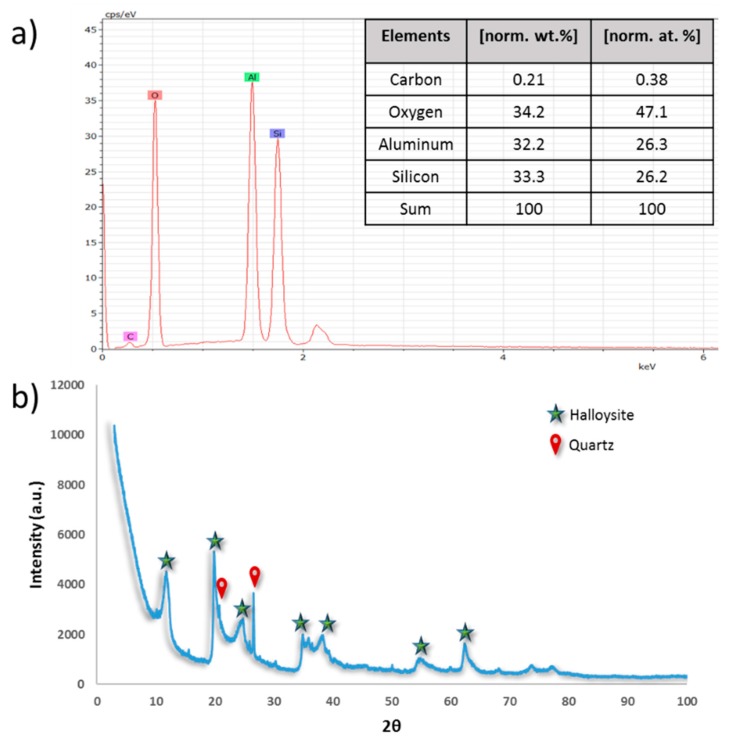
Elemental characterization using EDS (**a**), XRD at 2θ diffraction angle (**b**) for raw HNTs.

Chemical characterization of HNTs samples was carried out using energy-dispersive X-ray spectroscopy (EDS). The EDS spectrum of raw HNTs in Figure 6a shows strong peaks for O, Al, and Si elements, which confirms the chemical structure of HNTs with 1:1 Al to Si ratio.

Clay minerals, such as HNTs, are basically identified by their basal spacing of the layered silicate structures [33]. The XRD patterns of raw HNTs are presented in Figure 6b. Results indicated that the HNTs sample has characteristic peaks at 2θ diffraction angle at 11.8, 19.9, 24.7, 34.9, 38.35, 54.9, and 62.5°, which correspond to d spacing values of 7.5, 4.4, 3.6, 2.6, 2.4, 1.7, and 1.5 Å, respectively. The diffraction peak at 11.8°, which corresponds to basal spacing of 7.5 Å, is a characteristic of kaolin-type mineral and of dehydrated halloysite [72,73]. However, it was observed that there are other peaks at 20.77 and 26.6°, which correspond to some SiO_2_ present in the analyzed sample.

#### 3.1.3. Thermal Analysis 

**Figure 7:**
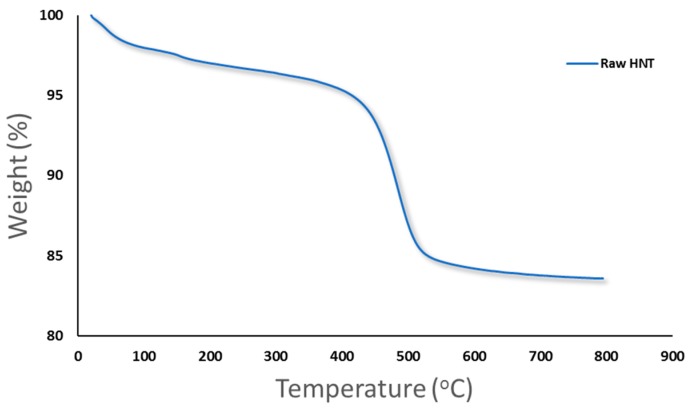
TGA analysis for raw HNTs.

Figure 7 shows the results of thermogravimetric (TGA) analysis for HNTs. It has been observed that there is a preliminary mass loss between 0 and 150 °C for the HNTs samples due to the loss of adsorbed water located in the surface and between the rolled sheets of HNTs (interlayer water) [53,63]. Another major weight loss that happened between 400 to 550 °C could be attributed to the dehydroxylation of the structural water present in the AlOH groups [74]. The total mass loss of HNTs at 800 °C was found to be 16.1% weight loss with a residue mass of 83.9%.

### 3.2. Membrane Characterization

#### 3.2.1. Membranes Morphology and Porous Structure 

The thickness of membrane samples was measured using a digital vernier clipper (Fowler, US) and found to be 80 μm for a PSF-based membrane matrix and range from 110 to 120 μm for a PSF/PVP-based membrane matrix, even though all membranes were casted at 200 μm gap height, as mentioned earlier in Section 2.2. The variation of the thicknesses between PSF and PSF/PVP-based membranes was probably because of the hydrophilic nature of PVP, which accelerates the instantaneous demixing of solvent/nonsolvent and therefore results in a fast polymer separation phase. It could be speculated that PVP as a hydrophilic copolymer causes a rapid formation of the top skin layer of the membrane at the beginning of phase inversion process [75] followed by a fast solvent/nonsolvent exchange rate, which may be the main reason for the increase in the membrane thickness observed in PSF/PVP based membranes. The fast leaching out of PVP from the membrane during the immersion precipitation process could be another reason for the increased thickness of PSF/PVP based membranes. 

**Figure 8:**
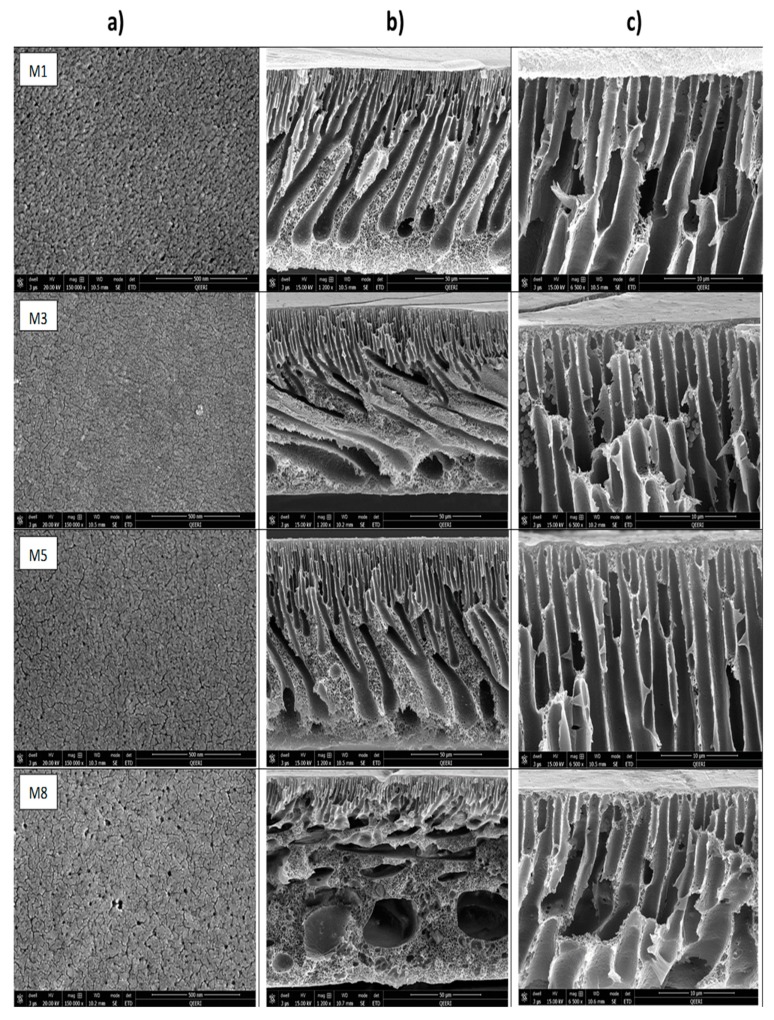

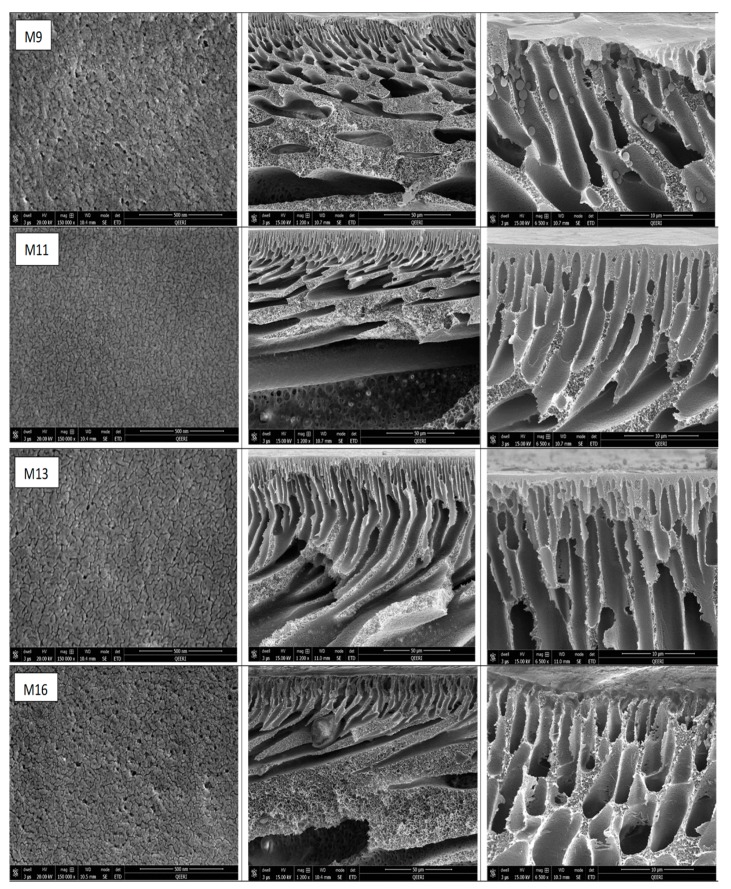
SEM images for: top surface (**a**), cross-section (**b**), enlarged top cross-section (**c**) for M_1_, M_3_, M_5_, M_8_, M_9_, M_11_, M_13_, M_16_ cast membranes.

**Figure 9:**
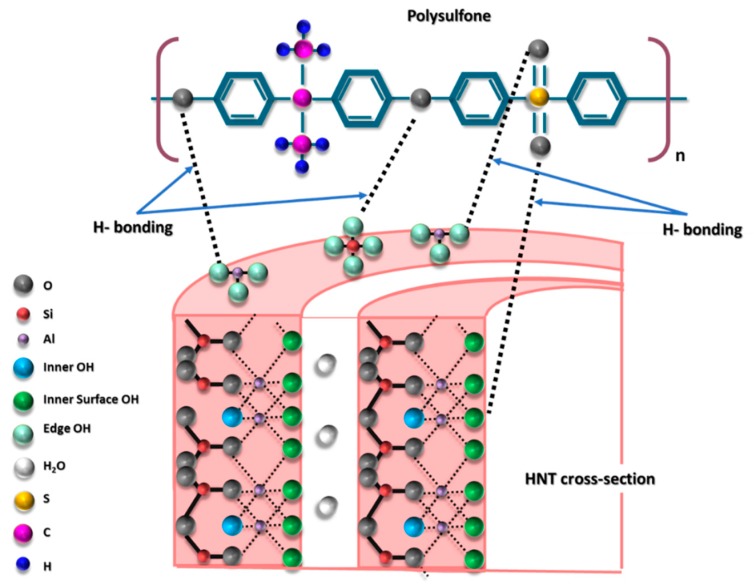
Schematic representation of probable sites of interactions between PSF and HNTs that could form hydrogen bonds.

Top surfaces and cross-sections morphologies of different fabricated membranes (M_1_, M_3_, M_5_, M_8_, M_9_, M_11_, M_13_, M_16_) are shown in Figure 8. As seen in this figure, the top surface of the neat PSF membrane (M_1_) has relatively larger pores compared with M_3_ and M_5_ samples. It seems that the addition of low amounts of HNTs of 0.2 or 0.5 wt % to the casting solution results in top surfaces that have denser but smaller pore sizes. These findings are somehow unexpected as the majority of the previous studies reported an increase in the membrane’s surface pore size upon the addition of hydrophilic nanoparticles to the casting solution, which results in a faster solvent/nonsolvent demixing during membrane formation [52,60]. Though the origin of decreasing membrane pore size at low HNTs loading compared to neat PSF membrane is still unclear, it might be assumed that at a low HNTs loading to the casting solutions, the gain in hydrophilicity has not been adequate enough to notably contribute into faster solvent/nonsolvent demixing during membrane formation. On the other hand, the decrease in the membrane pore size at low HNTs loading could be an indication of good compatibility and a strong interaction between PSF and HNTs. Due to the presence of hydroxyl groups located on HNTs surfaces, the nanotubes might form numerous hydrogen bonds with PSF, as shown in the schematic diagram represented in Figure 9. This could result in some slowing down of demixing rates between solvent and nonsolvent in the coagulation bath during the formation of the membrane’s top skin layer. The decrease in pore size on the top membrane’s surface was also observed in PSF/PVP membranes with 0.2 and 0.5 wt % HNTs loading (M_11_ and M_13_ samples in Figure 8). Similar observations were previously reported for the incorporation of CNTs and modified HNTs into PES and PSF membrane matrices, respectively [50,76]; however, these studies gave no explanation for such a phenomenon.

As seen in Figure 8, large pores were present on the top membrane surface when adding 5.0 wt % HNT to PSF and PSF/PVP casting solutions (M_8_ and M_16_ samples). This could be due to an essential increase of hydrophilicity of the casting solution at high HNT loading. As a result, a sharp increase in solvent/nonsolvent exchange takes place when the cast polymer film was immersed in the coagulation bath, which leads to fast polymer precipitation and formation of the enlarged pores in M_8_ and M_16_ membrane samples.

**Figure 10:**
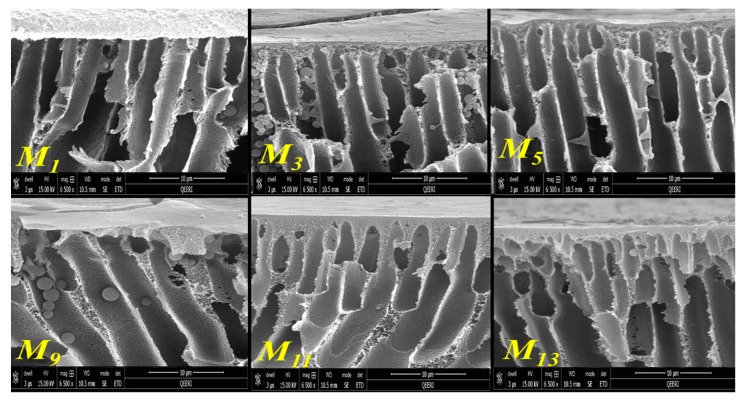
Zoomed SEM images of the skin top layer of M_1_, M_3_, M_5_, M_9_, M_11_, M_13_ membranes.

**Figure 11:**
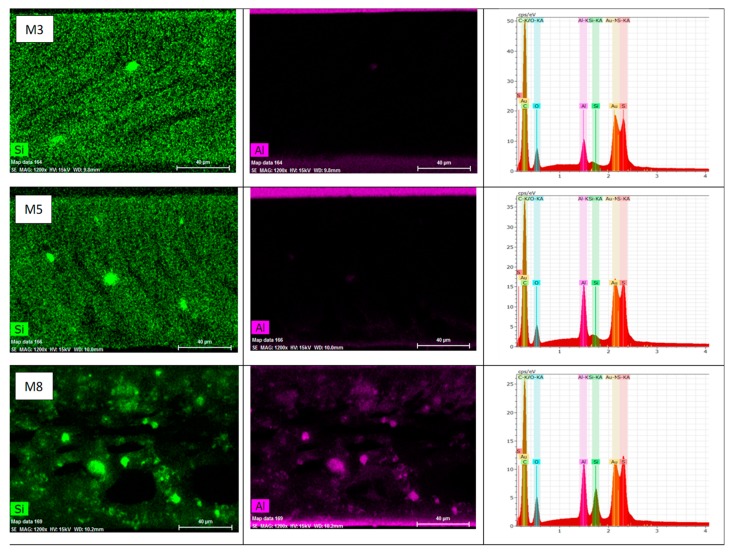
EDS images and elemental mapping of M1, M5, and M8 membranes.

The cross-section of the cast membranes shown in Figure 8b indicates that the prepared membranes show an asymmetric structure typical for ultrafiltration membranes and composed of distinct layers of different morphologies and pore sizes, including a thin top skin-layer (formed at the very beginning of phase inversion [77]), a sublayer with finger-like macro pores (formed just after the top skin layer) [77], and in some cases, a big macrovoids bottom layer [52]. The asymmetric cross-section morphology becomes somehow different after the incorporation of varying amounts of HNTs to both PSF (M_1_) and PSF/PVP (M_9_) membrane matrices. When adding low HNTs contents of 0.2 or 0.5 wt % to the PSF membrane matrix, the structure of the finger-like sublayer changed from tilted, long, and wide finger-like pores, which stops before reaching the bottom of the membrane in M_1_ sample, to a cross-section composed of two distinct finger-like structures: straight, short, and thin finger-like pores beneath the top skin layer and large, long, finger-like pores that extend to the bottom of the membrane in M_3_, M_5_ samples (Figure 8b). The changes in cross-section morphology observed in M_3_ and M_5_ samples could be due to slow formation of the top skin layer because of the interaction between HNTs and PSF (Figure 9), which would result in a smaller surface pore size (Figure 8a) and compacted skin top layer (Figure 8c and Figure 10). With the addition of 5.0 wt % HNTs to PSF (M_8_ sample), the thickness of the finger-like sublayer has been limited to a narrow stripe under the skin layer with a separate layer of macrovoids expanded from the middle to the bottom of the membrane. This observation could be explained by the fact that at high HNTs content of 5.0 wt %, the interaction between PSF and HNTs will be reduced significantly, and the number and size of HNTs aggregates will be enhanced (as seen in Figure 11). At this stage, the thermodynamic instability of the membrane system intensifies because of large amounts of hydrophilic HNTs. As a result, a rapid solvent/nonsolvent exchange rate took place, which leads to the creation of larger pores in the membrane’s surface and skin layer, as seen in Figure 8a,c (M_8_). Since in the phase inversion process the top skin layer is the first layer to be formed, these larger pores allow large volume of water to inter and form wider finger-like pores in the sub-layer. However, due to the higher viscosity of the casting solution of M_8_ that has 5.0 wt % HNTs content; the formation of appreciable number of HNTs aggregates (seen in Figure 11) that are concentrated on the middle and the bottom of the membrane, the solvent/nonsolvent exchange rate would be hindered below the membrane’s skin and sub-layer, which restrain the formation of long finger-like structure and limit it to the narrow thin sub-layer below the top skin layer. 

As seen in Figure 8, PVP addition to PSF casting solution has influenced the membrane porous structure (M_9_ sample). PVP, which has a high affinity to nonsolvent (water) used as a hydrophilic pore forming agent, is known to enhance the instantaneous demixing of the dope solution during the membrane formation process [78]. PVP is also known to influnce solvent’s dissolving capacity and improve solvency of polymer in the casting solution [23]. It can be seen in Figure 8a that the M_9_ sample has larger surface pores compared to the M_1_ sample, a short finger-like substructure, and a big macrovoid membrane’s middle and bottom structure that has been formed obviously due to the increased solvent/nonsolvent exchange rate during membrane casting. The surface of M_9_ resembles a carpet-like appearance due to fast leaching out of PVP during membrane formation. However, incorporation of HNTs in the PFS/PVP casting solution restrain this carpet-like surface structure. This observation suggests that HNTs may form hydrogen bonds with PVP, which restrict PVP leaching out of the membrane. Adding of both HNTs nanoparticle and PVP to the PSF casting solution results in their synergistic effect on the membrane’s morphology and porous structure.

As can be seen in Figure 8c and Figure 10, the top skin-layer in M_3_ and M_5_ are slightly thicker than in the M_1_ sample. As discussed earlier, this could be attributed to more homogenous distribution and strong interaction of HNTs with PSF matrix, which reduces the solvent/nonsolvent exchange rate when low HNTs contents were incorporated. On the other hand, the top skin-layer became thinner for the M_8_ sample that has 5.0 wt % HNTs contents. At high HNTs loading, the hydrophilicity of the casting solution increases, and hence, the solvent/nonsolvent exchange rate also increases, which results in a thinner top skin layer. For the PSF/PVP system in Figure 10, a thicker top skin-layer when adding low HNTs content (M_11_ and M_13_) and thinner top skin-layer when adding high HNTs content (M_16_) were also observed. Interestingly, at the same HNTs loading, the top membrane’s skin layer for M_11_ and M_13_ samples from the PSF/PVP matrix is thicker than the top skin layer for M_3_ and M_5_ from the PSF matrix. Probably, due to the formation of some hydrogen bonds between PVP and HNTs, PVP facilitates the uniform distribution of HNTs within the casting solution and, thus, improves the nanotubes interaction with the PSF matrix. 

The EDS analysis shown in Figure 11 depicts the localization and distribution of Al and Si elements, which are the main components of HNTs, within the PSF membrane matrix. HNTs aggregates can be identified in the membrane’s cross-section images by green and purple dot lights, which represent Si and Al elements, respectively. From Figure 11, it can be observed that HNTs disperse well in the PSF matrix at low HNTs loadings of 0.2 and 0.5 wt %, and only a few HNTs aggregates are visible in the M_3_ and M_5_ samples. However, at high HNTs content (M_8_ sample), HNTs tend to aggregate, obviously because of a high length-to-diameter ratio of the nanoparticles [62]. These aggregates are presumably localized at the membrane’s middle and bottom layers due to the relatively high weight and density of the HNTs nanoparticles. 

#### 3.2.2. Porosity and Pore Size 

**Figure 12:**
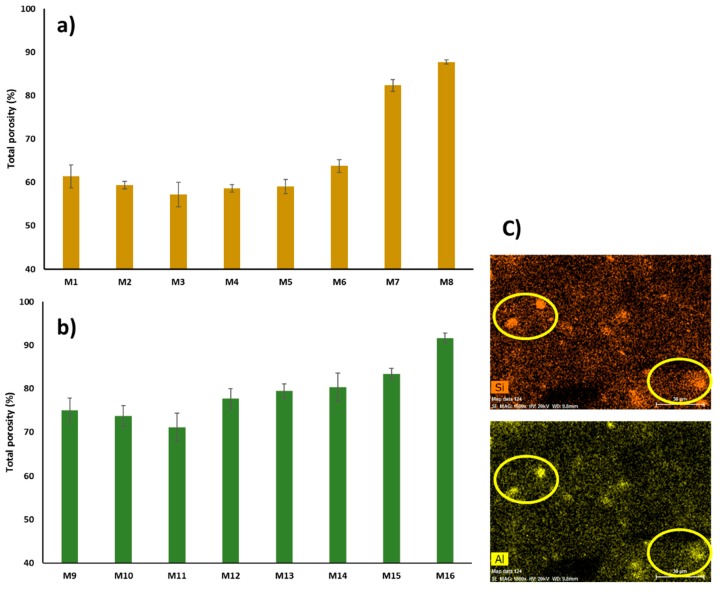
Total porosity for: PSF matrix (**a**), PSF/PVP matrix (**b**), EDS mapping analysis for M_16_ (**c**).

The porosity of the fabricated membranes was investigated using the gravimetric method mentioned in Section 2.3, and the results are displayed in Figure 12a,b. It can be observed from this Figure that there is a slight decrease in the total porosity when incorporating a low content of HNTs in the PSF casting solution. For example, the total porosity decreases from 61.4% for the pristine PSF membrane (M_1_) to 57.2% for PSF/0.2 wt % HNTs membrane (M_3_). As was discussed earlier, at low content, HNTs are well distributed within the PSF matrix. Strong interaction between PSF and HNTs nanoparticles might result in a decrease in the total membrane porosity. The obtained data of this decrease in porosity are consistent with other reports that incorporated hydrous ferric oxide modified HNTs [56] and dextran grafted HNTs into a PES membrane matrix [60]. The total membrane porosity started to rise when the HNTs content increased above 0.2 wt %, and the porosity reached the maximum value of 87.7% when the HNTs loading increased to 5.0 wt % in M_8_. The increase in total porosity observed in M_8_ could be attributed to the increase in numbers of hydroxylic groups present in HNTs, which attract water molecules (nonsolvent) and accelerate the solvent/nonsolvent exchange rate during phase inversion [65]. When high HNTs loadings were incorporated, the observed big macrovoids in the membrane’s middle and bottom layers and the presence of appreciable numbers of aggregates also contributed to the increased porosity in M_8_. These aggregates consisted of filler-to-filler networks (HNTs-to-HNTs networks) with free volume and a considerable number of OH^-^ functional groups that attract and facilitate the passage of water more than polymer-to-filler networks (PSF-to-HNTs) [46]. 

Figure 12b shows the effect of PVP incorporation in the PSF matrix on membrane porosity. As seen in this figure, total porosity increased from 61.4% for pure the PSF membrane (M_1_) to 75% for PSF/PVP membrane (M_9_). The increase in porosity could be explained by the fact that PVP acts as a nonsolvent swelling agent and, therefore, triggers the formation of larger network pores in the membrane’s skin and sublayer and bigger macrovoids in the middle and bottom layer [79,80]. Also, compared with PSF based membranes, PSF/PVP based membranes have larger membrane thickness, which would contribute to their overall porosity increase. With the incorporation of HNTs (up to 0.2 wt %) into the PSF/PVP membrane matrix, the total porosity decreased from 75.0% for the M_9_ sample to 71.1% for the M_11_ sample. The total membrane porosity started to rise when HNTs loading exceeded 0.2 wt %, and the porosity reached its maximum value of 91.6% for the M_16_ sample that has a 5.0 wt % HNTs content. The sharp increase in the total porosity observed in M_16_ could be due to the loose surface and cross-section structure that resulted from the incorporation of both HNTs and PVP hydrophilic additives to the PSF casting solution that sharply accelerated the solvent/nonsolvent exchange rate during membrane formation. In addition, the presence of HNTs aggregates in the M_16_ sample (Figure 12c) that have free volume and appreciable amounts of OH^−^ groups can facilitate the absorption of water within the aggregates. Surprisingly, when comparing EDS elemental mapping for the M_8_ sample in Figure 11 with the M_16_ sample in Figure 12c, it is seen that the light intensity for Si and Al elements in HNTs aggregates found in sample M_8_ is stronger than in M_16_, and a lesser number of HNTs aggregates was found in M_16_. This finding indicates that HNTs aggregation is more pronounced in the PSF membrane matrix than in the PSF/PVP matrix. Obviously because of their surface active properties, PVP macromolecules, which have carbonyl groups, can form hydrogen bonds with hydroxylic groups of the HNTs nanoparticles. As a result, PVP with its miscible affinity to nonsolvent and its tendency for leaching out of the membrane during membrane phase separation can reduce the HNTs aggregation and the formation of tightly packed HNTs aggregates. 

**Figure 13:**
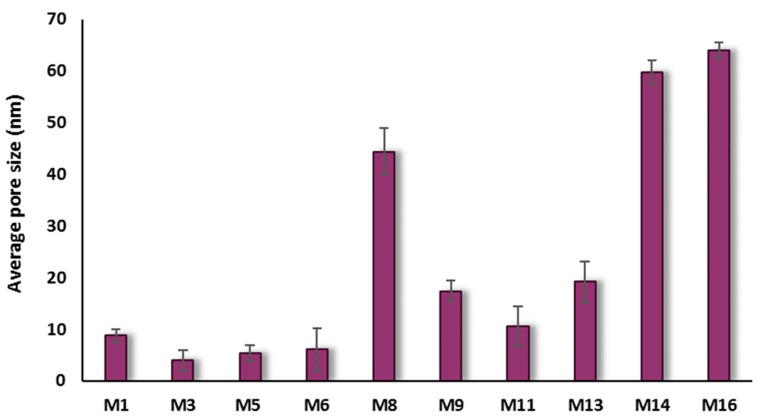
Pore size for some of PSF-HNTs and PSF/PVP-HNTs composite membranes.

Data on pore size of the prepared membranes are presented in Figure 13. As seen in this figure, the membrane’s surface pore size varies between 4.0 and 44.3 nm for PSF/HNTs nanocomposite-based membranes and between 10.6 and 64.0 nm for PSF/PVP/HNTs nanocomposite-based membranes. The membrane pore size decreases at low HNTs content when HNTs are more homogenously distributed within both PSF and PSF/PVP matrices (M_3_, M_5_, M_11_, and M_13_ samples). At moderate HNTs contents of 1.0 wt % in both PSF and PSF/PVP matrices, the average pore size for M_6_ sample is less than for M_1_, while it is more in M_14_ than in M_9_. It might be assumed that PVP interaction with HNTs facilitates the migration of nanoparticles toward the membrane surface, which results in faster solvent/nonsolvent exchange and the formation of larger surface pores observed in M_14_. A sharp increase in pore size was observed for M_8_ and M_16_ samples when high content of HNTs (5.0 wt %) was added to PSF and PSF/PVP matrices, respectively.

#### 3.2.3. Water Contact Angle

**Figure 14:**
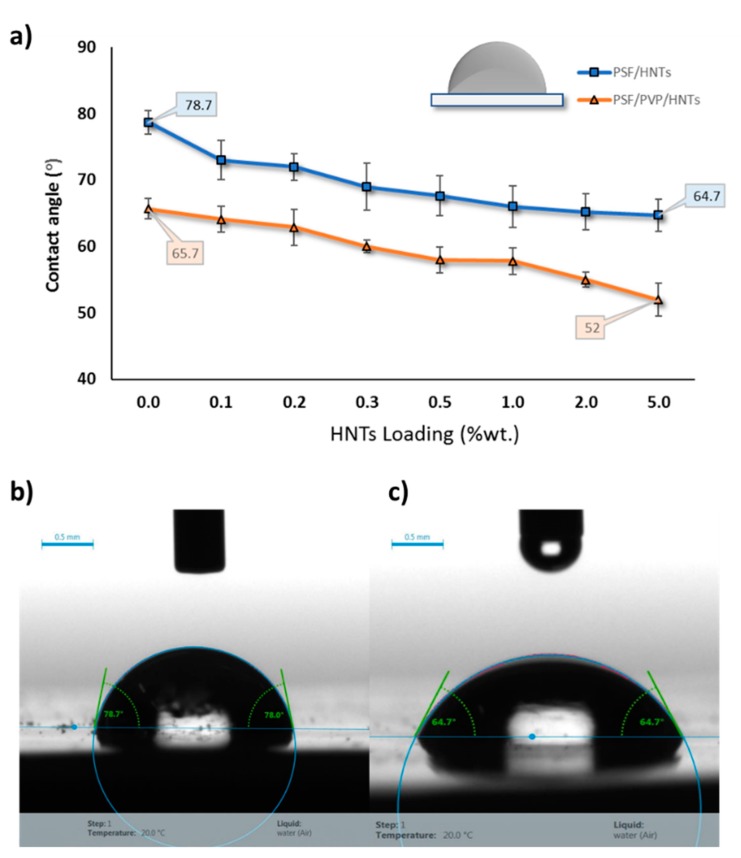
Water contact angle analysis for PSF/HNTs and PSF/PVP/HNTs membranes with different HNTs loading from 0.0 to 5.0 wt % (**a**), real water drop images for PSF membrane (**b**), and for PSF/5.0 wt % HNTs membrane (**c**).

Water contact angle (WCA) at the membrane surface determines the surface hydrophilicity and wettability of a membrane [81], and this is an important parameter for evaluating the membrane’s ability for fouling. An adjacent hydration layer is formed at a hydrophilic membrane surface, which behaves as a physical barrier for foulants to be deposited on the membrane’s surface [57,82]. Figure 14a shows the WCA values for PSF and PSF/PVP-based membranes at different HNTs loading. Because of the intrinsic hydrophobic nature of PSF, the WCA for pure PSF membrane (M_1_) showed the highest reading of 78.7° ± 1.7° among all cast membranes. It could be observed in Figure 14a that HNTs addition to the PSF casting solution notably reduced the WCA value from 78.7° ± 1.7° to 64.7° ± 2.5° when HNTs loading increased from 0 to 5.0 wt %. The decrease in WCA values for HNTs containing membranes is explained by the hydrophilic nature of HNTs additive, which contains a large number of hydroxylic groups [57]. 

For the PSF/PVP based membrane matrix, the addition of PVP to the casting solution decreased the WCA of the cast membranes from 78.7° ± 1.7° to 65.7° ± 1.6°. PVP is a water soluble copolymer additive, which causes swelling in the cast membrane and increases hydrophilicity [83]. The incorporation of HNTs into the PSF/PVP membrane matrix steadily reduces the WCA from 65.7° ± 1.6° for PSF/PVP membrane (M_9_) to 52° ± 2.5° for PSF/PVP/5.0 wt % HNTs (M_16_) membrane sample. The sharp decrease in WCA value for M_16_ can be attributed to increased membrane hydrophilic content as well as the increase in the membrane’s surface pore size, which allows water drops to penetrate quickly into the membrane’s pores. Figure 14b,c shows images of water drops captured at the surface of PSF (M_1_) and PSF/5.0 wt % HNTs (M_8_) membranes, respectively. 

#### 3.2.4. Mechanical Properties

**Figure 15:**
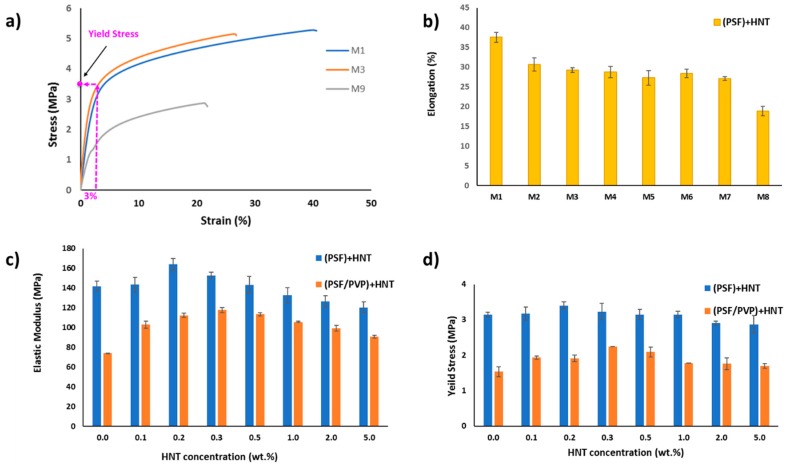
Stress–strain curves for M_1_, M_3_, and M_9_ (**a**), elongation at break for PSF/HNTs composite membranes (**b**), elastic modulus for all casted membranes (**c**), and yield stress for all casted membranes (**d**).

In pressure-driven membrane processes, the robust mechanical properties of the membrane are very important for durable long-term membrane performance. In this study, HNTs were used as a reinforcing nanoscale additive to improve the mechanical properties of PSF and PSF/PVP membrane matrices. Figure 15a depicts the stress–strain curves for M_1_, M_3_, and M_9_ membranes. As seen in the figure, all membrane samples exhibited an elastic deformation in the beginning of the stress–strain curves followed by plastic deformation. It also can be seen that there is a positive enhancement (an increase in the initial slope of the stress-strain curve) after adding a small amount of HNTs (0.2 wt %) to the PSF matrix in the M_3_ sample compared to M_1_ sample. Figure 15a also shows that by adding PVP to the PSF matrix; the stress–strain curve for the M_9_ (PSF/PVP) sample diminishes dramatically compared with the pure PSF matrix (M_1_). This is mainly because the PVP addition increases the membrane pore size and porosity, which weakens the mechanical properties. Figure 15b displays the effect of adding different HNTs loadings to the PSF membrane matrix on the elongation at break ((Δl/l)%, where l is the initial sample length). The pure PSF membrane sample (M_1_) exhibited the largest elongation of 38%, whereas the membrane sample with 5.0 wt % HNTs content (M_8_) showed the least elongation of 19%. This means that the addition of excessive HNTs contents to the PSF matrix reduces its flexibility. However, at low to moderate HNTs content (from 0.1 to 2.0 wt %), the decrease in membranes’ elongation at break values are relatively small compared to the pristine PSF membrane with values ranging between 31% and 27%. Despite the fact that, in many cases, the incorporation of nano-sized inorganic materials into a polymeric membrane matrix reduces its elongation at break (ductility/flexibility), this weak point can be neglected because, in real membrane application, the shear stress exerted by the feed solution flow onto the membrane surface is small [84]. The best mechanical properties (elastic modulus and yield stress) on each membrane matrix were achieved where low HNTs contents were incorporated into the PSF and PSF/PVP matrices. This is because the strong adhesion forces between HNTs and membrane matrices were attained due to good dispersion of HNTs within the polymer matrix, which is confirmed by EDS elemental mapping in Figure 11. Figure 15c,d shows the elastic modulus (the slope of the linear part of the stress-strain curve) and the yield stress (which is defined as the onset point of plastic yielding and was determined by the value of the stress which corresponds to 3% strain on the stress-strain curve) for all cast membranes. According to the obtained results, the incorporation of low content of HNTs into the PSF membrane matrix increases both the elastic modulus and yield stress with an optimal HNTs loading of 0.2 wt %. The elastic modulus increased by 15.6%, from 142 MPa in pristine PSF membrane to 164.1 MPa in the PSF/0.2 wt % HNTs sample. Also, the yield stress increased by 8%, from 3.15 MPa in pure PSF membrane to 3.4 MPa in PSF/0.2 wt % HNTs membrane. Incorporation of PVP in the PSF membrane matrix (M_9_) reduces the elastic modulus by 48% and the yield stress by 51% compared to M_1_. Although the obtained values of the elastic modulus and yield stress for PSF/PVP based membranes are lower compared to the values for PSF based membranes, the incorporation of 0.3 wt % of HNTs in the PSF/PVP matrix drastically elevate the elastic modulus and yield stress by 59% and 46.4%, respectively. The elastic modulus increased from 74.2 MPa for M_9_ to 117.8 MPa for M_12_, and the yield stress increased from 1.53 MPa for M_9_ to 2.24 MPa for M_12_. The increase in HNTs content, which gives the optimal elastic modulus and yield stress (from 0.2 wt % in PSF matrix to 0.3 wt % in PSF/PVP matrix) indicates that PVP enhances the distribution of HNTs within the membrane matrix. The overall notable increasing trend of the elastic modulus and yield stress at HNT content of 0.2 wt. % in PSF matrix and 0.3 wt % in PSF/PVP matrix could be ascribed to the high compatibility of HNTs within the PSF matrix due to the formation of hydrogen bonds with the polymer, as shown in Figure 9. Good HNTs distribution, as well as the interfacial interactions with PSF, dictate the HNTs reinforcing effect in PSF composite membranes, particularly at low HNT content (up to a loading of 0.2 wt % and 0.3 wt % in PSF and PSF/PVP matrices, respectively). However, the elastic modulus and yield stress decreased gradually when the HNTs loading increased above the optimal content in both PSF and PSF/PVP polymer matrices. These findings can be explained by HNTs aggregation at high HNTs loadings. The presence of HNTs aggregates causes weaker spots and inefficient stress transfer at the PSF-HNTs interface. However, when comparing elastic modulus and yield stress for the membrane with the weakest mechanical properties among the PSF membrane matrix (M_8_) with the membrane with the strongest mechanical properties among the PSF/PVP membrane matrix (M_12_), it is seen that the M_8_ sample is still higher by 2.4% and 28% in terms of elastic modulus and yield stress compared to the M_12_ membrane. 

### 3.3. Performance of PSF/HNTs and PSF/PVP/HNTs Membranes

#### 3.3.1. Pure Water Flux

**Figure 16:**
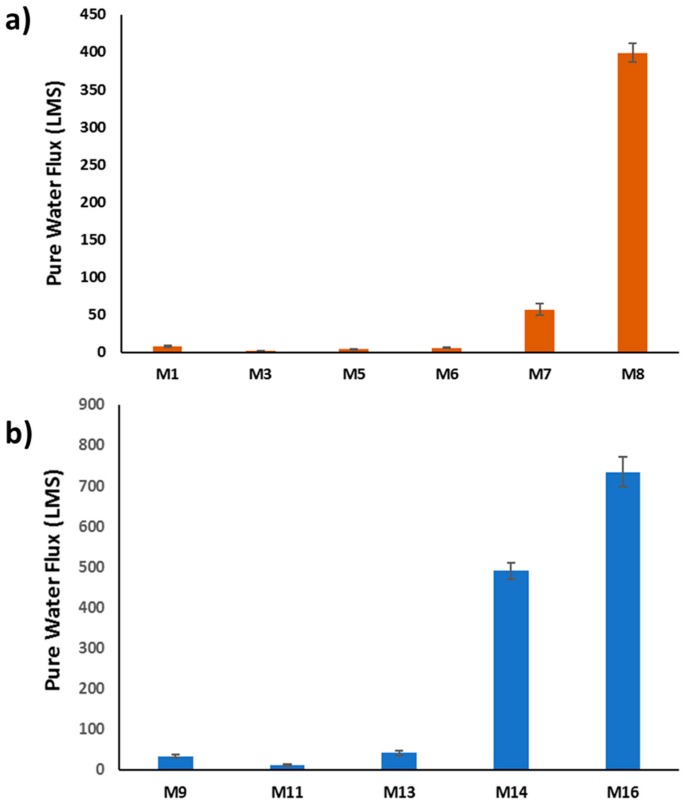
Pure water flux for some of PSF-HNTs composite membranes (**a**), PSF/PVP-HNTs composite membranes (**b**) (test conditions: DI water, 1 bar at room temperature, 30 min experimental duration time).

To investigate the effect of HNTs incorporation in both PSF and PSF/PVP matrices on membranes’ performance, filtration tests were conducted, as was described in Section 2. It is seen in Figure 16a,b that the pure water flux (PWF) values decreased at low HNT loading in both PSF and PSF/PVP membrane matrices. For example, the flux decreased from 7.8 LMH for the PSF membrane (M_1_) to 2.2 LMH for the PSF/0.2 wt % HNTs sample (M_3_) and from 32.8 LMH for the PSF/PVP membrane (M_9_) to 11.6 LMH for the PSF/PVP/0.2 wt % HNTs (M_11_) sample. This decrease in PWF values can be explained by the decrease in the membrane’s pore size and formation of thicker top skin membrane layers at low HNTs loadings, as was discussed in Section 3.2.1 and Section 3.2.2 As seen in Figure 16, PWF values for the cast membranes start to increase at HNT loading above 0.2 wt % for both PSF and PSF/PVP membrane matrices. A strike increase in PWF values was observed for both membrane matrices when HNTs content raised to 5.0 wt %: from 7.9 LMH for M_1_ to 398.9 LMH for M_8_, and from 32.8 LMH for M_9_ to 734.3 LMH for M_16_ samples. Such PWF increase could be attributed to larger pore size and total porosity, thinner top skin membrane layer, as well as higher hydrophilicity of PSF/5.0 wt % HNTs (M_8_) and PSF/PVP/5.0 wt % HNTs (M_16_) membranes. The M_16_ sample possesses the highest flux of all cast membranes of 734 LMH. The results of PWF are consistent with the results of membranes pore size and porosity.

#### 3.3.2. BSA Filtration Test 

**Figure 17:**
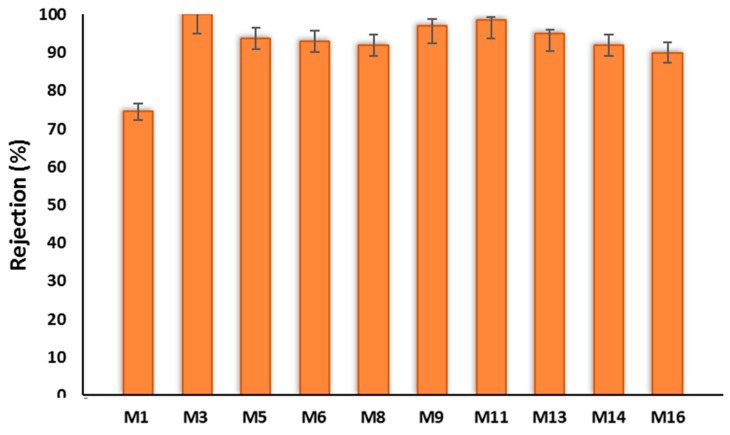
BSA rejection of PSF-HNTs and PSF/PVP-HNTs composite membranes (test conditions: filtration of 100 mg/L BSA solution at room temperature using 1 bar pressure, 30 min experimental duration time).

BSA rejection data for the prepared membranes are displayed in Figure 17. It can be seen from the figure that all membranes incorporated with HNTs and/or PVP had high BSA rejection (≥90%). The rejection for PSF based membranes follow the increasing order of M_1_, M_5_, M_6_, M_5_, and M_3_ and for PSF/PVP-based membranes, the increasing order of M_16_, M_14_, M_13_, M_9_, and M_11_. As can be seen, even with a high HNTs content of 5.0 wt % in the PSF membrane matrix, the M_8_ sample maintained a BSA rejection of 92%. The highest BSA rejection (almost 100%) was shown by the M_3_ membrane, obviously due to its smallest pore size compared to the other membrane samples. The high rejection values exhibited by the PSF/HNTs membranes could also be associated with the increase of membranes’ negative surface charge due to the incorporation of HNTs in the membrane matrix [85]. 

**Figure 18:**
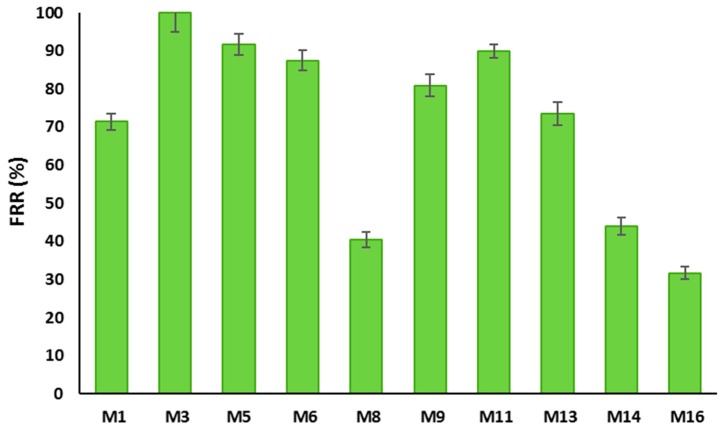
Effect of HNTs loading on flux recovery ratios (FRR) for some of as-prepared membranes evaluated for both PSF and PSF/PVP-based membranes.

Flux recovery ratio (FRR) is often used to evaluate the membrane’s anti-fouling properties [58]. FRR values for the prepared PSF and PSF/PVP-based membranes are displayed in Figure 18. It can be seen from this figure that the FRR values for PSF/HNTS membranes range from 40.5 to 100%, while it ranges from 31.7 to 89.9% for PSF/PVP/HNTs-based membranes. It is observed that incorporation of HNTs into both membrane matrices initially increased the FRR values and then a gradual decrease took place as the HNTs content increased. The M_3_ sample was able to restore 100% of the flux after being subjected to BSA filtration compared to 71.4% for the pristine membrane (M_1_). M_5_ and M_6_ also show higher recoveries of 91.7% and 84.6%, respectively, which are both higher than the recovery ratio for the plain PSF membrane (M_1_). This is obviously due to the improved hydrophilicity of the PSF/HNTs membrane samples. However, the FRR for the M_8_ sample has been reduced to 40.5% obviously due to its larger pore size, which allows BSA molecules to be absorbed and entrapped inside the membrane porous structure and within the HNTs aggregates [57]. The low FRR value for the M_8_ membrane is consistent with its relatively high rejection observed in Figure 17. The same trend in increasing FRR values at lower HNTs contents and decreasing FRR values at higher HNTs content was observed for PSF/PVP based membranes.

## 4. Conclusions

In the present study, novel PSF membranes incorporated with different HNTs loadings were synthesized via a phase inversion technique. Properties of the cast membranes were evaluated against a nascent PSF membrane. Characterization of HNTs confirmed that it has a nano-sized tubular structure with a well-defined empty lumen and open ends composed mainly of Si and Al elements. It was shown that upon the addition of a low HNTs content of 0.2 wt % to both PSF and PSF/PVP casting solutions, the membrane’s surface pore size and total porosity were decreased, and the thickness of the membrane’s skin layer was increased obviously due to the strong interaction between HNTs and PSF polymer matrix. Incorporation of HNTs content higher than 1.0 wt % lead to the formation of PSF/HNTs membranes that have a larger surface pore size, higher porosity, and thinner top-skin layer compared to the pristine PSF membrane. Some HNTs aggregates within the PSF membrane matrix were observed, especially at high 5.0 wt % of HNTs loading. WCA values showed that a membrane’s hydrophilicity was enhanced by the incorporation of HNTs to both PSF and PSF/PVP casting solutions. The mechanical performance test confirmed the compatibility and strong interfacial interaction between the HNTs and PSF matrix. The optimal HNTs loadings, which give the maximum elastic modulus and yield stress, were found to be 0.2 wt % for the PSF matrix and 0.3 wt % for the PSF/PVP matrix. It was found that the addition of PVP improves the HNTs distribution within the PSF/PVP polymer matrix and, hence, increased significantly the elastic modulus and yield stress of the prepared PSF/PVP-based membranes. However, PVP weakened the mechanical properties of the PSF matrix drastically. The BSA filtration tests showed that the PSF membrane incorporated with 0.2 wt % of HNTs showed almost 100% BSA rejection and possessed a high value of flux recovery.

## Figures and Tables

**Table 1:** Composition of synthesized polysulfone (PSF) membranes *.

Membrane	PVP Content (wt %)	HNT Content (wt %)
M1	-	-
M2	-	0.1
M3	-	0.2
M4	-	0.3
M5	-	0.5
M6	-	1.0
M7	-	2.0
M8	-	5.0
M9	1	-
M10	1	0.1
M11	1	0.2
M12	1	0.3
M13	1	0.5
M14	1	1.0
M15	1	2.0
M16	1	5.0

* All membranes were composed of 16 wt % PSF dissolved in 84 wt % *N*,*N*-dimethylacetamide (DMA) solvent.

## References

[1] Sri Abirami Saraswathi M.S., Rana D., Divya K., Gowrishankar S., Sakthivel A., Alwarappan S., Nagendran A. (2020). Highly permeable, antifouling and antibacterial poly (ether imide) membranes tailored with poly (hexamethylenebiguanide) coated copper oxide nanoparticles. Mater. Chem. Phys..

[2] Liu X., Yuan H., Wang C., Zhang S., Zhang L., Liu X., Liu F., Zhu X., Rohani S., Ching C. (2020). A novel PVDF/PFSA-g-GO ultrafiltration membrane with enhanced permeation and antifouling performances. Sep. Purif. Technol..

[3] Tian M., Wang R., Goto A., Mao W., Miyoshi Y., Mizoguchi H. (2020). Performance enhancement of ultrafiltration membrane via simple deposition of polymer-based modifiers. J. Water Process Eng..

[4] Harrigan D.J., Yang J., Sundell B.J., Lawrence J.A., O’Brien J.T., Ostraat M.L. (2020). Sour gas transport in poly (ether-b-amide) membranes for natural gas separations. J. Membr. Sci..

[5] Yin J., Zhang C., Yu Y., Hao T., Wang H., Ding X., Meng J. (2020). Tuning the microstructure of crosslinked Poly (ionic liquid) membranes and gels via a multicomponent reaction for improved CO2 capture performance. J. Membr. Sci..

[6] Tang S., Dong Z., Zhu X., Zhao Q. (2019). A poly (ionic liquid) complex membrane for pervaporation dehydration of acidic water-isopropanol mixtures. J. Membr. Sci..

[7] Wu G., Li Y., Geng Y., Jia Z. (2019). In situ preparation of COF-LZU1 in poly (ether-block-amide) membranes for efficient pervaporation of n-butanol/water mixture. J. Membr. Sci..

[8] Singh A.K., Kumar S., Bhushan M., Shahi V.K. (2020). High performance cross-linked dehydro-halogenated poly (vinylidene fluoride-co-hexafluoro propylene) based anion-exchange membrane for water desalination by electrodialysis. Sep. Purif. Technol..

[9] Beh J.J., Ooi B.S., Lim J.K., Ng E.P., Mustapa H. (2020). Development of high water permeability and chemically stable thin film nanocomposite (TFN) forward osmosis (FO) membrane with poly(sodium 4-styrenesulfonate) (PSS)-coated zeolitic imidazolate framework-8 (ZIF-8) for produced water treatment. J. Water Process Eng..

[10] Mahdi E.M., Tan J.-C. (2016). Mixed-matrix membranes of zeolitic imidazolate framework (ZIF-8)/Matrimid nanocomposite: Thermo-mechanical stability and viscoelasticity underpinning membrane separation performance. J. Membr. Sci..

[11] Manawi Y., Kochkodan V., Mohammad A.W., Ali Atieh M. (2017). Arabic gum as a novel pore-forming and hydrophilic agent in polysulfone membranes. J. Membr. Sci..

[12] Lavanya C., Soontarapa K., Jyothi M.S., Geetha Balakrishna R. (2019). Environmental friendly and cost effective caramel for congo red removal, high flux, and fouling resistance of polysulfone membranes. Sep. Purif. Technol..

[13] Rezaei-DashtArzhandi M., Sarrafzadeh M.H., Goh P.S., Lau W.J., Ismail A.F., Mohamed M.A. (2018). Development of novel thin film nanocomposite forward osmosis membranes containing halloysite/graphitic carbon nitride nanoparticles towards enhanced desalination performance. Desalination.

[14] Gao H., Sun X., Gao C. (2017). Antifouling polysulfone ultrafiltration membranes with sulfobetaine polyimides as novel additive for the enhancement of both water flux and protein rejection. J. Membr. Sci..

[15] Kang Y., Obaid M., Jang J., Ham M.-H., Kim I.S. (2018). Novel sulfonated graphene oxide incorporated polysulfone nanocomposite membranes for enhanced-performance in ultrafiltration process. Chemosphere.

[16] Wang K., Abdala A.A., Hilal N., Khraisheh M.K., Hilal N., Ismail A.F., Takeshi Matsuura T., Darren Oatley-Radcliffe D. (2017). Mechanical Characterization of Membranes. Membrane Characterization.

[17] Persson K.M., Gekas V., Trägårdh G. (1995). Study of membrane compaction and its influence on ultrafiltration water permeability. J. Membr. Sci..

[18] Katsoufidou K., Yiantsios S., Karabelas A. (2005). A study of ultrafiltration membrane fouling by humic acids and flux recovery by backwashing: Experiments and modeling. J. Membr. Sci..

[19] Pendergast M.M., Hoek E.M.V. (2011). A review of water treatment membrane nanotechnologies. Energy Environ. Sci..

[20] Wu H., Tang B., Wu P. (2010). Novel ultrafiltration membranes prepared from a multi-walled carbon nanotubes/polymer composite. J. Membr. Sci..

[21] Ciobanu G., Carja G., Ciobanu O. (2007). Preparation and characterization of polymer–zeolite nanocomposite membranes. Mater. Sci. Eng. C.

[22] Monticelli O., Bottino A., Scandale I., Capannelli G., Russo S. (2007). Preparation and properties of polysulfone–clay composite membranes. J. Appl. Polym. Sci..

[23] Chang X., Wang Z., Quan S., Xu Y., Jiang Z., Shao L. (2014). Exploring the synergetic effects of graphene oxide (GO) and polyvinylpyrrodione (PVP) on poly (vinylylidenefluoride) (PVDF) ultrafiltration membrane performance. Appl. Surf. Sci..

[24] Ma Y., Shi F., Zhao W., Wu M., Zhang J., Ma J., Gao C. (2012). Preparation and characterization of PSf/clay nanocomposite membranes with LiCl as a pore forming additive. Desalination.

[25] Shawky H.A., Chae S.-R., Lin S., Wiesner M.R. (2011). Synthesis and characterization of a carbon nanotube/polymer nanocomposite membrane for water treatment. Desalination.

[26] Wei Y., Chu H.-Q., Dong B.-Z., Li X., Xia S.-J., Qiang Z.-M. (2011). Effect of TiO2 nanowire addition on PVDF ultrafiltration membrane performance. Desalination.

[27] (2019). Ihsanullah Carbon nanotube membranes for water purification: Developments, challenges, and prospects for the future. Sep. Purif. Technol..

[28] Li S., Liao G., Liu Z., Pan Y., Wu Q., Weng Y., Zhang X., Yang Z., Tsui O.K.C. (2014). Enhanced water flux in vertically aligned carbon nanotube arrays and polyethersulfone composite membranes. J. Mater. Chem. A.

[29] Majumder M., Chopra N., Andrews R., Hinds B.J. (2005). Nanoscale hydrodynamics: Enhanced flow in carbon nanotubes. Nature.

[30] McGinnis R.L., Reimund K., Ren J., Xia L., Chowdhury M.R., Sun X., Abril M., Moon J.D., Merrick M.M., Park J. (2018). Large-scale polymeric carbon nanotube membranes with sub–1.27-nm pores. Sci. Adv..

[31] Garcia-Garcia D., Ferri J.M., Ripoll L., Hidalgo M., Lopez-Martinez J., Balart R. (2017). Characterization of selectively etched halloysite nanotubes by acid treatment. Appl. Surf. Sci..

[32] Tian F., Cui D., Schwarz H., Estrada G.G., Kobayashi H. (2006). Cytotoxicity of single-wall carbon nanotubes on human fibroblasts. Toxicol. In Vitro.

[33] Tharmavaram M., Pandey G., Rawtani D. (2018). Surface modified halloysite nanotubes: A flexible interface for biological, environmental and catalytic applications. Adv. Colloid Interface Sci..

[34] Goda E.S., Yoon K.R., El-sayed S.H., Hong S.E. (2018). Halloysite nanotubes as smart flame retardant and economic reinforcing materials: A review. Thermochim. Acta.

[35] Liu M., Jia Z., Jia D., Zhou C. (2014). Recent advance in research on halloysite nanotubes-polymer nanocomposite. Prog. Polym. Sci..

[36] Marney D.C.O., Russell L.J., Wu D.Y., Nguyen T., Cramm D., Rigopoulos N., Wright N., Greaves M. (2008). The suitability of halloysite nanotubes as a fire retardant for nylon 6. Polym. Degrad. Stab..

[37] Jiang J., Zhang Y., Cao D., Jiang P. (2013). Controlled immobilization of methyltrioxorhenium (VII) based on SI-ATRP of 4-vinyl pyridine from halloysite nanotubes for epoxidation of soybean oil. Chem. Eng. J..

[38] Almasri D.A., Saleh N.B., Atieh M.A., McKay G., Ahzi S. (2019). Adsorption of phosphate on iron oxide doped halloysite nanotubes. Sci. Rep..

[39] Tan D., Yuan P., Liu D., Du P. (2016). Surface Modifications of Halloysite. Developments in Clay Science.

[40] Mu K., Zhang D., Shao Z., Qin D., Wang Y., Wang S. (2017). Enhanced permeability and antifouling performance of cellulose acetate ultrafiltration membrane assisted by l -DOPA functionalized halloysite nanotubes. Carbohydr. Polym..

[41] Smoot J. An Analysis on Using L-Dopa for ADHD. https://smartdrugsforcollege.com/l-dopa-for-adhd/.

[42] Zhu L., Wang H., Bai J., Liu J., Zhang Y. (2017). A porous graphene composite membrane intercalated by halloysite nanotubes for efficient dye desalination. Desalination.

[43] Hebbar R.S., Isloor A.M., Zulhairun A.K., Sohaimi Abdullah M., Ismail A.F. (2017). Efficient treatment of hazardous reactive dye effluents through antifouling polyetherimide hollow fiber membrane embedded with functionalized halloysite nanotubes. J. Taiwan Inst. Chem. Eng..

[44] Ibrahim G.P.S., Isloor A.M., Moslehyani A., Ismail A.F. (2017). Bio-inspired, fouling resistant, tannic acid functionalized halloysite nanotube reinforced polysulfone loose nanofiltration hollow fiber membranes for efficient dye and salt separation. J. Water Process Eng..

[45] Ghanbari M., Emadzadeh D., Lau W.J., Riazi H., Almasi D., Ismail A.F. (2016). Minimizing structural parameter of thin film composite forward osmosis membranes using polysulfone/halloysite nanotubes as membrane substrates. Desalination.

[46] Swapna V.P., Saranya E.P., Nithya A.B., Sabu T., Ranimol S. (2016). Properties of Polysulfone/Halloysite Nanocomposite Membranes: Prepared by Phase Inversion Method. Macromol. Symp..

[47] Zhu J., Guo N., Zhang Y., Yu L., Liu J. (2014). Preparation and characterization of negatively charged PES nanofiltration membrane by blending with halloysite nanotubes grafted with poly (sodium 4-styrenesulfonate) via surface-initiated ATRP. J. Membr. Sci..

[48] Zeng G., He Y., Zhan Y., Zhang L., Shi H., Yu Z. (2016). Preparation of a Novel Poly (vinylidene fluoride) Ultrafiltration Membrane by Incorporation of 3-Aminopropyltriethoxysilane-Grafted Halloysite Nanotubes for Oil/Water Separation. Ind. Eng. Chem. Res..

[49] Xu H., Li D., Liu Y., Jiang Y., Li F., Xue B. (2019). Preparation of halloysite/polyvinylidene fluoride composite membrane by phase inversion method for lithium ion battery. J. Alloy. Compd..

[50] Wang Y., Zhu J., Dong G., Zhang Y., Guo N., Liu J. (2015). Sulfonated halloysite nanotubes/polyethersulfone nanocomposite membrane for efficient dye purification. Sep. Purif. Technol..

[51] Guo X., Fan S., Hu Y., Fu X., Shao H., Zhou Q. (2019). A novel membrane biofouling mitigation strategy of D-amino acid supported by polydopamine and halloysite nanotube. J. Membr. Sci..

[52] Zeng G., Ye Z., He Y., Yang X., Ma J., Shi H., Feng Z. (2017). Application of dopamine-modified halloysite nanotubes/PVDF blend membranes for direct dyes removal from wastewater. Chem. Eng. J..

[53] Chen Y., Zhang Y., Zhang H., Liu J., Song C. (2013). Biofouling control of halloysite nanotubes-decorated polyethersulfone ultrafiltration membrane modified with chitosan-silver nanoparticles. Chem. Eng. J..

[54] Yu L., Zhang Y., Zhang H., Liu J. (2015). Development of a molecular separation membrane for efficient separation of low-molecular-weight organics and salts. Desalination.

[55] Moslehyani A., Mobaraki M., Ismail A.F., Matsuura T., Hashemifard S.A., Othman M.H.D., Mayahi A., Rezaei DashtArzhandi M., Soheilmoghaddam M., Shamsaei E. (2015). Effect of HNTs modification in nanocomposite membrane enhancement for bacterial removal by cross-flow ultrafiltration system. React. Funct. Polym..

[56] Wan Ikhsan S.N., Yusof N., Aziz F., Misdan N., Ismail A.F., Lau W.-J., Jaafar J., Wan Salleh W.N., Hayati Hairom N.H. (2018). Efficient separation of oily wastewater using polyethersulfone mixed matrix membrane incorporated with halloysite nanotube-hydrous ferric oxide nanoparticle. Sep. Purif. Technol..

[57] Mishra G., Mukhopadhyay M. (2018). Enhanced antifouling performance of halloysite nanotubes (HNTs) blended poly (vinyl chloride) (PVC/HNTs) ultrafiltration membranes: For water treatment. J. Ind. Eng. Chem..

[58] Buruga K., Kalathi J.T., Kim K.-H., Ok Y.S., Danil B. (2018). Polystyrene-halloysite nano tube membranes for water purification. J. Ind. Eng. Chem..

[59] Hebbar R.S., Isloor A.M., Inamuddin, Abdullah M.S., Ismail A.F., Asiri A.M. (2018). Fabrication of polyetherimide nanocomposite membrane with amine functionalised halloysite nanotubes for effective removal of cationic dye effluents. J. Taiwan Inst. Chem. Eng..

[60] Yu H., Zhang Y., Sun X., Liu J., Zhang H. (2014). Improving the antifouling property of polyethersulfone ultrafiltration membrane by incorporation of dextran grafted halloysite nanotubes. Chem. Eng. J..

[61] Zhang J., Zhang Y., Chen Y., Du L., Zhang B., Zhang H., Liu J., Wang K. (2012). Preparation and Characterization of Novel Polyethersulfone Hybrid Ultrafiltration Membranes Bending with Modified Halloysite Nanotubes Loaded with Silver Nanoparticles. Ind. Eng. Chem. Res..

[62] Zeng G., He Y., Zhan Y., Zhang L., Pan Y., Zhang C., Yu Z. (2016). Novel polyvinylidene fluoride nanofiltration membrane blended with functionalized halloysite nanotubes for dye and heavy metal ions removal. J. Hazard. Mater..

[63] Chen Y., Zhang Y., Liu J., Zhang H., Wang K. (2012). Preparation and antibacterial property of polyethersulfone ultrafiltration hybrid membrane containing halloysite nanotubes loaded with copper ions. Chem. Eng. J..

[64] Wang Z., Wang H., Liu J., Zhang Y. (2014). Preparation and antifouling property of polyethersulfone ultrafiltration hybrid membrane containing halloysite nanotubes grafted with MPC via RATRP method. Desalination.

[65] Zeng G., He Y., Yu Z., Zhan Y., Ma L., Zhang L. (2016). Preparation and characterization of a novel PVDF ultrafiltration membrane by blending with TiO2-HNTs nanocomposites. Appl. Surf. Sci..

[66] Babu V.S., Padaki M., D’Souza L.P., Déon S., Geetha Balakrishna R., Ismail A.F. (2018). Effect of hydraulic coefficient on membrane performance for rejection of emerging contaminants. Chem. Eng. J..

[67] Lim S., Park M.J., Phuntsho S., Mai-Prochnow A., Murphy A.B., Seo D., Shon H. (2018). Dual-layered nanocomposite membrane incorporating graphene oxide and halloysite nanotube for high osmotic power density and fouling resistance. J. Membr. Sci..

[68] Luo C., Zou Z., Luo B., Wen W., Li H., Liu M., Zhou C. (2016). Enhanced mechanical properties and cytocompatibility of electrospun poly (l-lactide) composite fiber membranes assisted by polydopamine-coated halloysite nanotubes. Appl. Surf. Sci..

[69] Li L., Wang F., Lv Y., Liu J., Zhang D., Shao Z. (2018). Halloysite nanotubes and Fe3O4 nanoparticles enhanced adsorption removal of heavy metal using electrospun membranes. Appl. Clay Sci..

[70] Rezaee R., Nasseri S., Mahvi A.H., Nabizadeh R., Mousavi S.A., Rashidi A., Jafari A., Nazmara S. (2015). Fabrication and characterization of a polysulfone-graphene oxide nanocomposite membrane for arsenate rejection from water. J. Environ. Health Sci. Eng..

[71] Sabri S., Najjar A., Manawi Y., Eltai N., Al-Thani A., Atieh M., Kochkodan V. (2019). Antibacterial Properties of Polysulfone Membranes Blended with Arabic Gum. Membranes.

[72] Brindley G. (1952). Identification of clay minerals by X-ray diffraction analysis. Clays and Clay Miner..

[73] Bordeepong S., Bhongsuwan D., Pungrassami T., Bhongsuwan T. (2011). Characterization of halloysite from thung yai district, Nakhon Si Thammarat Province, in Southern Thailand. Songklanakarin J. Sci. Technol..

[74] Brindley G.W. (1957). The Role of Water Vapour in the Dehydroxylation of Clay Minerals. Clay Miner..

[75] Kim N., Kim C.-S., Lee Y.-T. (2008). Preparation and characterization of polyethersulfone membranes with p-toluenesulfonic acid and polyvinylpyrrolidone additives. Desalination.

[76] Wang Y., Ou R., Ge Q., Wang H., Xu T. (2013). Preparation of polyethersulfone/carbon nanotube substrate for high-performance forward osmosis membrane. Desalination.

[77] Wijmans J.G., Baaij J.P.B., Smolders C.A. (1983). The mechanism of formation of microporous or skinned membranes produced by immersion precipitation. J. Membr. Sci..

[78] Chakrabarty B., Ghoshal A.K., Purkait M.K. (2008). Preparation, characterization and performance studies of polysulfone membranes using PVP as an additive. J. Membr. Sci..

[79] Qin J.-J., Wong F.-S., Li Y., Liu Y.-T. (2003). A high flux ultrafiltration membrane spun from PSU/PVP (K90)/DMF/1,2-propanediol. J. Membr. Sci..

[80] Sivakumar M., Mohan D.R., Rangarajan R., Tsujita Y. (2005). Studies on cellulose acetate-polysulfone ultrafiltration membranes: I. Effect of polymer composition. Polym. Int..

[81] Lalia B.S., Kochkodan V., Hashaikeh R., Hilal N. (2013). A review on membrane fabrication: Structure, properties and performance relationship. Desalination.

[82] Liu Z., Mi Z., Jin S., Wang C., Wang D., Zhao X., Zhou H., Chen C. (2018). The influence of sulfonated hyperbranched polyethersulfone-modified halloysite nanotubes on the compatibility and water separation performance of polyethersulfone hybrid ultrafiltration membranes. J. Membr. Sci..

[83] Basri H., Ismail A.F., Aziz M. (2011). Polyethersulfone (PES)–silver composite UF membrane: Effect of silver loading and PVP molecular weight on membrane morphology and antibacterial activity. Desalination.

[84] Homayoonfal M., Mehrnia M.R., Rahmani S., Mohades Mojtahedi Y. (2015). Fabrication of alumina/polysulfone nanocomposite membranes with biofouling mitigation approach in membrane bioreactors. J. Ind. Eng. Chem..

[85] Pasbakhsh P., Churchman G.J., Keeling J.L. (2013). Characterisation of properties of various halloysites relevant to their use as nanotubes and microfibre fillers. Appl. Clay Sci..

